# The Role of Health Departments in Advancing a New Community Health and Wellness Agenda

**Published:** 2007-06-15

**Authors:** Ellen Jones, Ellen Boyce, Joan Stine

**Affiliations:** National Association of Chronic Disease Directors; Office of Professional Practice, Region 1, South Carolina Department of Health and Environmental Control, Greenwood, SC; Center for Health Promotion, Education, and Tobacco Use Prevention, Maryland Department of Health and Mental Hygiene, Baltimore, Md

The National Expert Panel on Community Health Promotion provided recommendations that are relevant not only to federal agencies such as the Centers for Disease Control and Prevention (CDC) but also to state and local health agencies (1). State and local health agencies have a strong foundation in convening nontraditional partners, coordinating public and private efforts, and serving as a model for accountability and stewardship. These skills can be used to apply the principles outlined by the expert panel, including expanding surveillance systems to include social determinants of health and developing programs within the context of local culture and political processes.

Public health agencies must begin to develop surveillance measures of the socioecological determinants of health by seeking expertise in sociology, economics, and other sciences. With expanded surveillance measures, we in public health can determine how social factors influence prevention and management of chronic diseases. We can develop methods to define, quantify, measure, and weigh these socioecological indicators and establish benchmarks for these determinants of health and disease. This information will be critical to the design of effective strategies that target the social precursors of disease and to the measurement of the impact of these strategies on both social and health variables. Some communities, such as King County, Washington, have included social and health indicators in tracking the well-being of residents and improvements in community health (2).

State and local health agencies also have a role in developing public health programs in collaboration with community members and stakeholders and in coordinating these efforts to directly address community health concerns. As outlined in the expert panel report, true community engagement is needed to sustain chronic disease prevention activities over time. This approach is also evident in the tool *Mobilizing for Action through Planning and Partnerships* developed by the National Association of County and City Health Officials in cooperation with the Public Health Practice Program Office, CDC (3). Evidence linking socioecological factors to health will help bring new partners and community participation to public health when it becomes clear that our interests overlap. New surveillance tools will help us recognize social and ecological problems, predict their public health impact, and plan for long-term public health solutions involving both the public and private sectors. The expected improvement in community health can contribute to higher education levels, improved productivity, and improved earning capacity.

As health departments balance their focus on immediate health concerns with long-term community health, they will need to build the skills of an interdisciplinary workforce to meet the public health challenges of the future. The Institute of Medicine's report *Who Will Keep the Public Healthy?* promotes an ecological approach to addressing public health challenges in the 21st century (4). It states that public health professionals must have a framework for action, an understanding of the forces that affect health, and a model of health that emphasizes the linkages and relationships among multiple determinants affecting health. It goes on to recommend that public health agencies assess their workforce development needs, collaborate with schools of public health, and assure that their leadership has experience in the ecological approach to public health.

The expert panel made a strong recommendation to the National Center for Chronic Disease Prevention and Health Promotion to support training and capacity building to ensure that the public health workforce has the knowledge, skills, and tools to implement community health promotion approaches. These capacities may include sustainability, program evaluation, and socioecological dimensions of health. The Directors of Health Promotion and Education and the National Association of Chronic Disease Directors are devoting significant resources to assess training needs, provide relevant training opportunities, and expand the focus of community health training to address this recommendation.

Many public health agencies are taking advantage of new technologies to develop their existing workforce. Distance learning via satellite transmissions, webcasts, self-instruction using DVDs, and other new methods has provided access to quality continuing education even in rural areas. Public health agencies need to ensure that application of the socioecological model is included in determining the capacity of their workforce and that trainings are offered to build skills in using the model. Public health agencies must also disseminate best practices and lessons learned in applying these skills through traditional and nontraditional means as part of the larger framework of public health workforce development.
